# Population Structure Analysis of the Border Collie Dog Breed in Hungary

**DOI:** 10.3390/ani9050250

**Published:** 2019-05-16

**Authors:** Virág Ács, Árpád Bokor, István Nagy

**Affiliations:** 1 Department of Animal Sciences, Kaposvár University, Kaposvár, H-7400, 40, Guba S. str., Hungary; nagy.istvan@ke.hu; 2 Department of Hippology, Kaposvár, Kaposvár University, Kaposvár, H-7400, 40, Guba S. str., Hungary; bokor.arpad@ke.hu

**Keywords:** Border Collie, inbreeding, pedigree analysis, population size, subpopulations

## Abstract

**Simple Summary:**

The appearance of dog breeds is constantly changing, for many reasons. The Border Collie breed has several lines depending on sport, show, or work requirements, with closed breeding practices within these lines in recent decades. The aim of the study was to map the current population in Hungary and determine the possible inbreeding levels in and between the different subpopulations. The main finding of the study was that there is a detectable genetic divergence between the show and working line. In addition, genetic variability within the breed is decreasing due to a lack of suitable mating plans and the education of the breeders who are repeatedly choosing to breed animals with similar show-related characteristics. The size of the active breeding population has decreased dramatically in the past years. However, there are many dogs in the country without a pedigree. It can be seen that despite the proportion of registered breeders, dog owners prefer not to buy purebred dogs, and thus most of the pups born in Hungary are exported to other countries.

**Abstract:**

Pedigree data of the Border Collie dog breed were collected in Hungary to examine genetic diversity within the breed and its different lines. The database was based on available herd books dating from the development of the breed (in the late 1800s) to the present day. The constructed pedigree file consisted of 13,339 individuals, of which 1566 dogs (born between 2010 and 2016) composed the alive reference population which was active from breeding perspective. The breed is subdivided by phenotype, showing a thicker coat, harmonic movement, a wide skull, and heavier bones for the show type, and a thinner or sometimes short coat and smaller body for the working line, while the mixed line is quite heterogeneous (a combination of the above). Thus, the reference population was dissected according to the existing lines. The number of founders was 894, but eight individuals were responsible for contributing 50% of the genetic variability. The reference population had a pedigree completeness of 99.6% up to 15 generations and an inbreeding coefficient of 9.86%. Due to the changing breed standards and the requirements of the potential buyers, the effective population size substantially decreased between 2010 and 2016. Generation intervals varied between 4.09 and 4.71 years, where the sire paths were longer due to the later initial age of breeding in males compared to females. Genetic differences among the existing lines calculated by fixation indices are not significant; nonetheless ancestral inbreeding coefficients are able to show contrasts.

## 1. Introduction

The Border Collie is considered as one of the most intelligent dog breeds, and originated from the England Northumbria region. The breed’s name is related to the words “border” (between England and Scotland), “coal” (basic color), and “koolie” (useful) [1,2]. Breeding of the Collie-type dogs began in the British Islands in the 19th century in order to help the shepherds in gathering, driving, singling, penning, loading in trailers, and shedding races. Thus, the main objectives for breed selection, in descending order, were herding instinct, acceptance of human guiding, and speed. The name “Border Collie” was first used by the International Sheep Dog Society (ISDS) in 1915 in order to distinguish this breed from the Smooth and Bearded Collies, which at the beginning had similar breeding aspects. From the 1860s exhibitions became more and more popular, and their appearance was more consistent. The breed was registered by the British Kennel Club. The first public sheep herding event took place at Wales in 1873, where Old Hemp, one of the founders of the breed, appeared. Contrary to his conspecifics, his herding work was performed without barking, carefully approaching the sheep and controlling the flock with an intensive stare, or “eye”. These characteristics were defining the basics of today’s collecting style herding. 

The Border Collie breed was officially recognized by the *Féderation Cynologique Internationale* (*FCI*) in 1977, and the valid official breed standard was published in 1987 when selection for appearance began. Obtaining the status “show champion” was however only possible after passing the so-called working exam in several countries. 

Regarding the Hungarian Border Collie population, the first two females were imported in the country in 1995. The present population is not consistent; the number of registered breeders is 154. The Border Collie dogs are bred based on a closed herd book which predisposes to an increased inbreeding level of the population. It is also a common practice for dog breeders to choose sires and dams with a common ancestor to fix some specific traits in the appearance or working ability. This so-called line breeding tries to avoid closely-related mating; however, the breeding association sometimes permits it. The use of specific lines within one breed was previously reported in France [3], where more than 49% of the breeders claimed to use line breeding in practice. There are three specific lines within the breed worldwide, which are also represented in Hungary, such as the show line, the working line, and the so-called “mixed” line originating from Australia and New-Zealand. This “mixed” line combines the appearance of show dogs (heavier bones, elegant head, thicker coat) with the energy and stamina of the working-lined dogs, with the objective of use in search and rescue, therapy, or sports.

In case of working and show dogs, there are slight differences among breed standards between countries; thus, import of breeding animals is not always appropriate from the breeder’s point of view. Maintaining genetic diversity in order to create productive and healthy offspring without increasing the genetic load is the key to maintaining small populations. Hence, the objective of this study was to analyze the effects of closed breeding on the population structure of the Border Collie dog breed in Hungary and examine the genetic diversity within and between the subpopulations. 

## 2. Materials and Methods

The pedigree dataset of the Hungarian Border Collie population was constructed using available electronic herd books and pedigrees from Hungarian breeders. These websites could be searched but their content could not be downloaded, and therefore all genealogy data had to be retyped manually. First, the reference population was defined as the living and active animals (from the perspective of breeding) born between 2010 and 2016. The reference population was composed of 1566 dogs (703 males and 863 females). Then, the available genealogy information of these dogs was traced back and was recorded, creating the pedigree of the whole population from the late 1800s to the present day. The created Hungarian Border Collie pedigree dataset contained 13,339 individuals (5649 males and 7750 females). 

The genealogy records used in this study were created using the software “Equihun Pedigree Builder” [4]. The following information was entered:

- Individual identity number
- Male parent
- Female parent
- Date of birth
- Country code (i.e., county of origin)
- Color (not used in this study)
- Sex

After exporting the pedigree records their correctness was checked, pedigree analysis was performed applying ENDOG software [5]. The structure of the Hungarian Border Collie population was characterized by the following parameters:

- Number of founders (f: ancestors with two unknown parents)
- Effective number of founders (fe: the number of equally contributing founders that would be expected to produce the same genetic diversity as in the population under study)
- Effective number of ancestors (fa: similar to fe but replacing the contributions of founders with the marginal contributions of ancestors)
- Number of ancestors responsible for 50% of the genetic variability (fa50)
- Generation interval (average age of parents at the birth of their progeny kept for reproduction)
- Pedigree completeness (proportion of its known ancestors per generation)
- Inbreeding coefficient (the probability that the two alleles at any locus in an individual are identical by descent)
- Average relatedness (the probability that an allele randomly chosen from the whole population belongs to a given animal)
- Effective populations size (realized effective population size) from an individual increase of inbreeding [6].

All these parameters were explained in detail by [5]; therefore equations related to the listed parameters will not be repeated here. 

Then, the database was dissected into three groups. The inbreeding coefficients were also adjusted in the three lines. In addition, fixation indices (F_IS_, F_ST_) were calculated to detect the reduction of heterozygosity among subpopulations and individuals for measuring the total population differentiation [7]. 

For the calculation of the fixation indices, coancestry and kinship distance were used [8,9], using the following equations:

(1) $$F_{IS}=\frac{\bar{F}-\bar{f}}{1-\bar{f}}$$

(2) $$F_{ST}=\frac{\bar{f}-\bar{f}}{1-\bar{f}}=\frac{\bar{D}}{1-\bar{f}}$$

where $\bar{f}$ is the mean coancestry for the metapopulation; $\bar{F}$ is the mean inbreeding coefficient for the metapopulation; and $\bar{D}$ is the average genetic distance between the subpopulations.

Moreover, ancestral inbreeding coefficients proposed by the authors of [10,11,12] and determined with GRain 2.0 software [13] were used to obtain whether these distinct measurements for inbreeding are able to describe the differences between the lines. For the determination of the cumulative proportion of the genome that was exposed to inbreeding effects, ancestral inbreeding (F__BAL_) was calculated in the subpopulations. In addition, inbreeding coefficient was also calculated by the method of [11] by dividing inbreeding into two parts, based on whether part of the identical alleles were inbred in the past (F__KAL_) or became inbred in recent generations (F__KAL_NEW_). 

## 3. Results 

### 3.1. The Probability of Gene Origin

Trends in the probability of gene origin fe, fa50, and their ratios are presented in Table 1.

Compared to a great number of founders, a large part of the genetic variability is maintained based on only eight ancestors (Table 1). Looking at the observed ratio of fa and fe it can be concluded that the Hungarian Border Collie population has suffered very strong gene loss.

### 3.2. Effective Population Size

**Figure 1:**
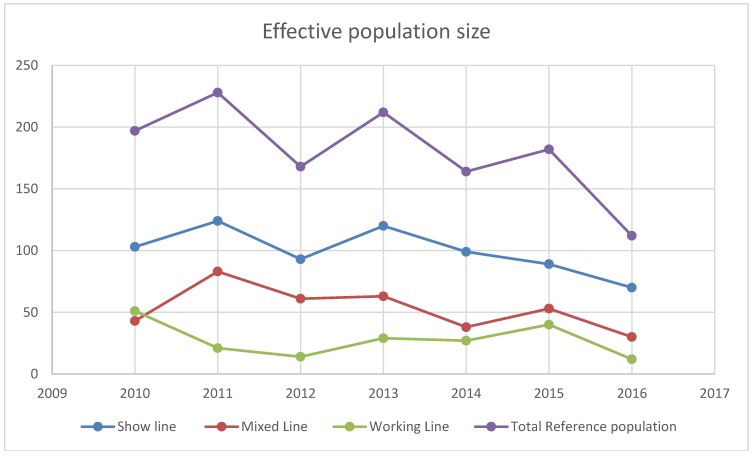
Effective population size (Ne) of the reference population.

Trends in the realized effective population size are presented in Figure 1. 

Due to the unequal contribution of the breeding animals to the next generation, the effective population size is always smaller compared to the exact population size. Unfortunately, this decreasing trend may coincide with the loss of genetic variability and with the appearance of genetic diseases [14]. Similar tendencies were reported in several dog breeds [15].

### 3.3. Generation Interval

The generation interval was calculated as the average age of parents at the birth of their progeny kept for the reproduction, and it was computed for all four parent–progeny pathways (Table 2) represented in the total reference population and per line. 

The length of the generation interval (T) can be substantially divergent across different breeds. In the present study, the sire paths were longer as the males were kept in breeding for longer ages than females and there is also a tendency for breeders to prefer to use males with more show and sport results rather than males with few titles even if they could increase the genetic variability within the breed. Collection of titles requires many years, so the preferred breeding males are usually older than the females. Intervals within the show and working lines were similar; however, the mean age of the father when the offspring was born was somewhat lower in the mixed line (sire–son path: 2.71 years, sire–daughter path: 4.33 years).

### 3.4. Inbreeding and Average Relatedness

Evolution of the inbreeding coefficient and the average relatedness of the reference population is provided in Table 3. 

As the population size of the Border Collie breed in Hungary is relatively large, the increase of the inbreeding level was relatively small (10% per 23 years). The decrease of the average inbreeding coefficients between 2011 and 2014 can be explained by the intense import of breeding animals. Because inbreeding coefficients are always dependent on the length and on the completeness of the pedigree the inbreeding coefficients were plotted on the complete generation equivalents [16]. Ancestral inbreeding coefficients were added to determine if inbred alleles in the past may have influenced the characterisations of these different phenotypes (Table 4). 

Adding ancestral inbreeding coefficients, Ballou’s formula showed that individuals in the working line had less probability of inheriting an allele which had undergone inbreeding in the past at least once than individuals in the show and the mixed lines. When estimating the proportion of each dog’s genome that was identical by descent in an ancestor to alleles identical by descent for the first time in that dog’s linage by the gene dropping method, the F__KAL_ and F__KAL_NEW_ showed similar results. Calculations for ancestral inbreeding were previously used by the authors of [10,11,12]. 

The differences between the show and working lines regarding Ballou’s formula are 20.4%; in addition the working line also differs from the mixed line by 20%. Furthermore, both Kalinowski’s formulas show that the working line suffered less inbreeding in the past few generations.

In the total population, only 2.77% of the matings were highly inbred (0.16% between full-sibs, 1.74% between half-sibs, and 0.87% between parent–offspring). Inbreeding coefficients differed among the studied lines; the average inbreeding coefficient was 4.9% in the reference population of the working line, while it reached 10.51% and 11.03% between 2010 and 2016 in the mixed and show lines, respectively. 

**Figure 2:**
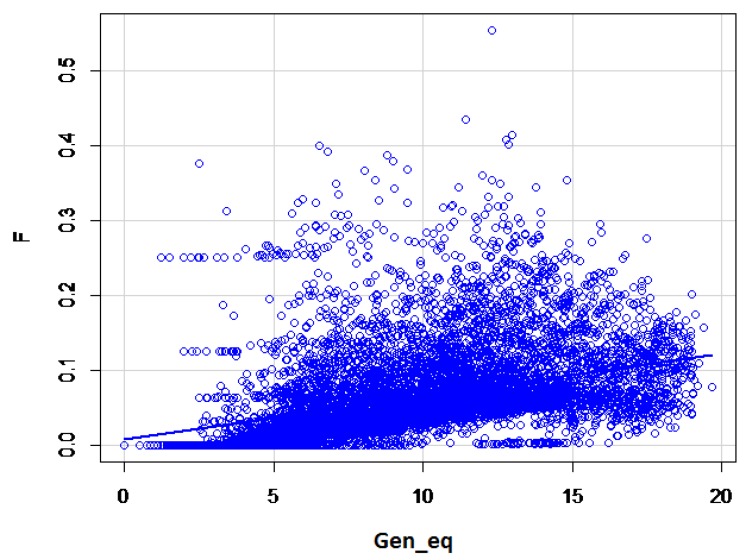
Association between inbreeding coefficient and complete generation equivalents in the total population. F: inbreeding coefficient; Gen_eq: number of complete generation equivalents.

The maximum and average number of complete generation equivalents were 25.04 and 4.47, respectively. A slow but continuous increase of the inbreeding coefficient based on the increasing complete generation equivalent was obvious (Figure 2). For the first 15 generations, the pedigree completeness was 99.6%, decreasing to 87.6% by the 40th generation. 

### 3.5. Fixation Indices in Subpopulations

In the calculation of the subdivision of the lines (Table 5), the within-variety fixation index (F_IS_) was 0.36%, showing that mating within lines was not random; this is in contrast to sheep and horse breeds, where this value is negative [17], showing that individuals in farm animal species are less related. In the reference population (fa/fe ratio: 0.17), the overall F_ST_ was 2.6%, with decreasing heterozygosity at the subpopulation level; however the genetic differences are still not significant, despite the diversity of the pheotype of the lines in the past 20 years. Analogous fixation indices were measured studying native Italian hunting dog breeds with microsatellite markers [3].

**Figure 3:**
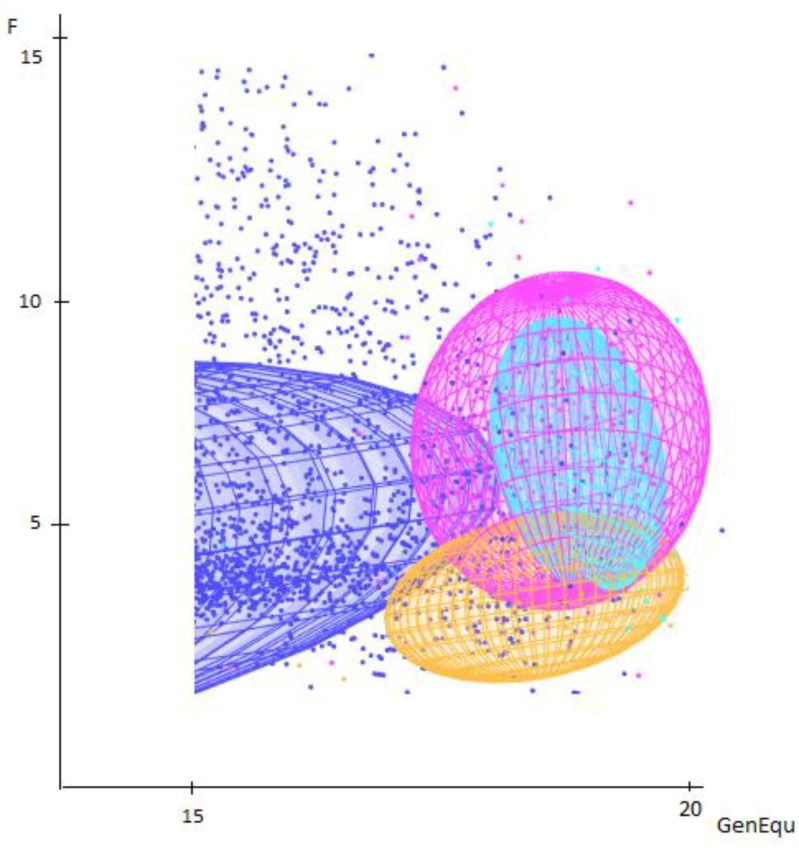
The inbreeding coefficients of the different lines depending on the total generation equivalent, where F is the inbreeding coefficient of an individual and GenEqu is the number of complete generation equivalents. 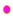 Show line, 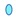 Mixed line, 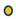 Working line, 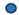 Total population.

Figure 3. represents the effects to the subdivision on the reference population, highlighting that the studied lines started to separate; nonetheless this is not statistically proven.

## 4. Discussion 

In many populations, all estimates related to the probability of gene origin decreased the most during the first years. Unfortunately, in this study, the analyzed period covered more than a century. Therefore, determining annual numbers was not possible. Similar findings were also reported in the French Beauceron and Braque Francais dog populations [18,19]. The presence of preferential breeding can be shown by calculating the ratio of fe and fa. Small values signal the so-called bottleneck effect [20]. If fe is larger compared to fa, the population suffers from gene loss and consequently a decrease in genetic variation [16]. Comparing this result to other populations, the medium value (0.75) of the fa/fe ratio of the Braque Francais dog population shows a more balanced use of animals for breeding and an absence of a bottleneck in that population [18]. The observed small ratio of fa and fe of the Hungarian Border Collie population can be explained by its closed herd book, intense selection for appearance, and by the favoritism of some relevant individuals

One of the reasons for decreasing the effective population size is that most of the puppies born in Hungary are sold abroad due to the lack of suitable owners for the breed. However, the dog-keeping culture is improving; there are still many backyard breeders who are selling puppies at often half of the cost of the breeder’s price, resulting in a huge border collie mix population. Besides, accommodating to the breed standards also decreases the effective population size. 

For the generation intervals, the results are not surprising as the reproductive life of sires is usually longer compared to dams. Similar results were found in the Nova Scotia Duck Tolling Retriever and the Lancashire Heeler dog breeds [21], and for several French dog breeds [18,19]. The lower length of the mixed-line generation interval can be attributed to the fact that these dogs are bred with lower show and working performance. 

In dog breeding, mating of close relatives is a common practice, where the objective is to create an outstanding individual [22]; however, after few generations, the raised inbreeding level escalates juvenile mortality [23]. Moreover, this non-random mating is able to increase inbreeding depression (bringing alleles to a homozygous state), affecting the future genetic health of the breed.

For pedigree completeness, the obtained values show that the available electronic herd books register all ancestors, and that the Hungarian Border Collie population has an exceptionally long and complete pedigree. This pedigree quality is only comparable to that of thoroughbred horses and Pannon White rabbit populations [24,25].

## 5. Conclusions

Since the final objectives for dogs in shows and sports require different anatomical structures, the importance of these lines is outstanding. However, the contrasts in dog selection may increase the genetic distance. In the long run, continuous selection for different purposes such as for show and work may disrupt the breed. The decreasing tendency of the effective population size points out a trend that dog owners prefer not to buy from registered breeders. In Hungary, the working line is at the greatest risk in terms of the number of breeding animals and the number of litters. However, this line represents the look and the original function of the breed. To maintain variability, the genetic contribution of some preferred males could be limited by mating schemes in order to help the breeders. Importation of breeding dogs could be a solution to this problem; on the other hand, breeding standards are slightly different between countries, and thus a collaboration is required between breeding organizations and scientists to improve the health of the next generation. 

## Figures and Tables

**Table 1:** Demographic parameters of the border collie breed in Hungary.

f	894
fg	806
fe	117
fa	20
fa50	8
fe/fa ratio	0.13
fa/fe ratio	0.17

f: number of founders; fg: equivalent number of founders; fe: effective number of founders; fa: effective number of ancestors; fa50: number of ancestors responsible for 50% of the genetic variability.

**Table 2:** Descriptive statistics of the generation interval (T) for the reference population (2010–2016).

Pathway	*N*	T	SD	SE
Father–Son	67	4.43	2.63	±0.341
Father–Daughter	107	4.71	2.64	±0.335
Mother–Son	67	4.17	1.65	±0.220
Mother–Daughter	106	4.09	1.63	±0.217
	Show line			
Father-Son	13	4.84	2.93	±0.813
Father–Daughter	25	5.01	3.12	±0.865
Mother–Son	13	4.41	1.81	±0.504
Mother–Daughter	25	4.22	2.10	±.584
	Mixed line			
Father–Son	7	2.71	0.92	±0.348
Father–Daughter	15	4.33	1.95	±0.738
Mother–Son	7	3.29	1.60	±0.607
Mother–Daughter	15	3.44	1.25	±0.472
	Working line			
Father–Son	14	3.42	1.24	±0.717
Father–Daughter	26	4.22	1.87	±1.08
Mother–Son	14	4.34	1.38	±0.80
Mother–Daughter	26	4.48	2.07	±0.158

*N*: number of individuals in the reference population; T: generation interval; SD: standard deviations; SE: standard errors of means.

**Table 3:** Inbreeding (F) and average relatedness (AR) of the reference population.

Birth Year	*N*	F	F__BAL_	F__KAL_	F__KAL_NEW_	AR
2010	269	8.71	61.1	7.97	1.98	7.38
2011	245	9.32	61.4	7.39	1.92	7.39
2012	199	10.6	61.9	8.83	2.33	7.32
2013	233	10.5	61.9	8.69	2.14	7.11
2014	188	9,3	62.3	7.98	1.75	7.23
2015	214	10.1	59.9	8.26	1.92	7.06
2016	219	10.5	50.3	4.56	1.59	7.42

*N*: Number of individuals; F: inbreeding coefficient; F_BAL: Ballou’s formula for ancestral inbreeding; F_KAL: Kalinowski’s formula for ancestral inbreeding; F_KAL_NEW: Kalinowski’s new formula to ancestral inbreeding; AR: average relatedness.

**Table 4:** Ancestral inbreeding coefficients in subpopulations (%).

Birth Year	*N*	F	F__BAL_	F__KAL_	F__KAL_NEW_	AR
**Show line**						
2010	104	9.67	63.70	7.93	1.73	7.30
2011	119	9.50	60.40	7.61	1.94	7.70
2012	93	11.90	62.30	9.38	2.58	7.17
2013	120	12.40	63.50	10.00	2.35	7.06
2014	99	10.80	65.30	9.05	1.77	7.23
2015	101	11.30	64.60	9.75	2.45	7.00
2016	100	11.70	63.90	9.60	2.10	7.20
**Mixed line**						
2010	43	11.50	62.60	8.94	2.63	7.37
2011	70	10.20	61.70	7.82	2.18	7.55
2012	52	12.10	63.20	8.91	2.40	7.39
2013	63	10.50	62.80	7.64	2.85	7.31
2014	38	8.79	63.10	7.26	1.53	7.27
2015	61	10.00	64.50	9.77	1.73	7.31
2016	-	-	-	-	-	-
**Working line**						
2010	51	5.69	54.20	4.35	1.43	7.50
2011	12	4.62	53.60	3.82	0.63	7.63
2012	14	5.18	53.70	4.80	0.3	7.67
2013	29	4.60	52.40	5.14	0	7.14
2014	27	6.00	51.50	4.33	0.17	7.22
2015	40	4.68	45.30	3.66	0.10	6.45
2016	12	3.53	43.90	2.57	0.09	6.46

*N*: Number of individuals; F: inbreeding coefficient; F_BAL: Ballou’s formula to ancestral inbreeding; F_KAL: Kalinowski’s formula to ancestral inbreeding; F_KAL_NEW: Kalinowski’s new formula to ancestral inbreeding; AR: average relatedness.

**Table 5:** Genetic similarities within the population.

Mean coancestry within Subpopulations F_ij_	0.079
Mean self-coancestry (s_i_)	0.541
Dij	0.024
F_ST_	0.026
F_IS_	0.0036

Fij: mean coancestry within subpopulations; si: self-coancestry; Dij: Nei’s minimum distance; FST: inbreeding caused by the differentiation of the subpopulation; FIS: inbreeding coefficients within the subpopulations.
